# Alleviation of Psychological Distress and the Improvement of Quality of Life in Patients With Amyotrophic Lateral Sclerosis: Adaptation of a Short-Term Psychotherapeutic Intervention

**DOI:** 10.3389/fneur.2018.00231

**Published:** 2018-04-16

**Authors:** Moritz Caspar Franz Oberstadt, Peter Esser, Joseph Classen, Anja Mehnert

**Affiliations:** ^1^Department of Neurology, University Medical Center Leipzig, Leipzig, Germany; ^2^Department of Medical Psychology and Medical Sociology, University Medical Center Leipzig, Leipzig, Germany

**Keywords:** amyotrophic lateral sclerosis, psychotherapeutic intervention, calm, quality of life, distress

## Abstract

Amyotrophic lateral sclerosis (ALS) is a progressive neurodegenerative disease that is inevitably fatal. To be diagnosed with a terminal illness such as ALS deeply affects one’s personal existence and goes along with significant changes regarding the physical, emotional, and social domains of the patients’ life. ALS patients have to face a rapidly debilitating physical decline which restrains mobility and impairs all activities of daily living. This progressive loss of autonomy may lead to a sense of hopelessness and loss of quality of life, which in turn may even result in thoughts about physician-assisted suicide. Here, we would like to propose a psychotherapeutic manualized, individual, semi-structured intervention to relieve distress and promote psychological well-being in ALS patients. This short-term intervention was originally developed for advanced cancer patients. “Managing Cancer and Living Meaningfully (CALM)” focuses on the four dimensions: (i) symptom management and communication with healthcare providers, (ii) changes in self and relations with close others, (iii) spirituality, sense of meaning and purpose and (iv) thinking of the future, hope, and mortality. We suggest to supplement the concept by two additional dimensions which take into account specific issues of ALS patients: (v) communication skills, and (vi) emotional expression and control. This therapeutic concept named “*M*anag*I*ng *B*urden in *A*LS and *L*iving *M*eaningfully (mi-BALM)” may be a further treatment option to help improving quality of life of ALS patients.

## Amyotrophic Lateral Sclerosis (ALS)

Amyotrophic lateral sclerosis represents a rapidly progressing neurodegenerative disease and is characterized by a degeneration of motor neurons (1). ALS affects the upper motor neurons projecting from the cortex to the brainstem and the spinal cord as well as the lower motor neurons projecting from the brainstem or spinal cord to the muscles (1). The initial presentation of ALS varies considerably between patients: while some patients first experience muscle weakness in the limbs, referred to as spinal onset, others initially suffer from dysarthria and dysphagia, characterized as bulbar onset. Bulbar onset appears in about one-third of patients with ALS and is often associated with emotional lability, which may arise from disconnection of brainstem structures from cortical inhibition (2, 3). Limb-onset weakness accounts for 60% of cases, presents usually asymmetrically and may first develop in either the upper or lower limbs. Apart from weakness, additional symptoms may include spasticity as a sign for upper motor neuron loss, and fasciculations, cramps and muscle atrophy as signs for lower motor neuron loss. Death usually results from respiratory failure which is caused by the loss of nerve supply of the respiratory muscles (3). The average life expectancy of patients with ALS is 2–3 years from the onset of symptoms, while a minority (4%) survive for 10 years or even longer (4, 5). The incidence of ALS is about two cases per 100,000 individuals and the age of onset peaks at 70–74 years (6).

In most cases, ALS appears to develop sporadically, although some patients have a familial disease which is associated with mutations in genes that have a wide range of functions (3). The primary symptoms of ALS are associated with motor dysfunction, such as muscle weakness or dysphagia, but more than 40% of ALS patients additionally develop cognitive or behavioral symptoms in advanced stage of the disease and about 14% of patients present with accompanying frontotemporal dementia (7).

## Mental Burden and Challenges in the Progression of the Disease

To be diagnosed with a life-threatening illness, such as ALS, deeply affects one’s personal existence and involves a variety of changes in the physical, emotional, and mental-cognitive aspects of a patients’ life. At first, ALS patients realize the debilitating physical symptoms due to muscle weakness impairing mobility and autonomy in all activities of daily living (8). A characteristic of ALS is the impairment to communicate verbally due to dysarthria, which means a motor disorder of speech characterized by abnormalities of the articulation and reduced intelligibility of speech. Dysarthria appears in 25–30% of ALS patients as a first or predominant sign in early stage (9) and the potential loss of speech has been rated as one of the three worst aspects of the disease by ALS patients (10). Furthermore, patients suffer from numerous symptoms, including pain, spasticity, difficulty in swallowing, weight loss, and respiratory insufficiency that require intensive treatment by a multidisciplinary care team (11). Among the wide range of symptoms, pain seems to be particularly frequent and to play a major role in affecting the quality of life in ALS patients (12, 13). Moreover, progressive muscle weakness leads to dependence on others like familial caregivers and/or a multiprofessional care team because of immobility (8). Patients have to face mental-cognitive challenges, to adapt to the new life situation and to decide about permanent and invasive medical measures including gastrostomy tube placement and assisted ventilation during disease progress (14). With the respect to the progredient loss of the ability to speak, patients and their related persons have to find new ways of communication, such as computer-based communication devices, such as eye-tracking systems (15). Progressive loss of autonomy and control may lead to severe emotional reactions, including of help- and hopelessness and a complete loss of motivation (16). Regarding potential risk factors for emotional impairments caused by the disease, hopelessness in ALS patients is predicted by the belief that life is determined by forces beyond his or her own control (external locus of control) and a lack of meaning of life, but not by socioeconomic or demographic factors, length or severity of illness, social support satisfaction or spiritual belief (17). Just as hopelessness, depressive symptoms are not related to time since diagnosis, the degree of disability or the progression of the illness (18), but slightly increase with speed of disease progression (19). Depression scores vary considerably between studies and range from 35 to 57% (20–24). Compared to other somatic patient groups including patients with cancer or heart failure, ALS patients most frequently ask for physician-assisted suicide (25, 26). Emotion in ALS patients may be difficult to recognize because emotional expression is frequently altered by the disease. Up to 49% of ALS patients show uncontrollable outbursts of laughter or crying without any appropriate environmental trigger and may be either more pronounced or even incongruent with the underlying emotional state (27, 28). This emotional expression disorder is named “pseudobulbar affect” and poses a problem in correct recognition and interpretation of emotional signs, not only for related persons, but also in therapeutic settings (29).

Taken together, previous research indicates a broad spectrum of physical, emotional, and mental-cognitive challenges for ALS patients (Table 1), which all have to be addressed by a multimodal (psycho-)therapy.

**Table 1:** Specific aspects of amyotrophic lateral sclerosis (ALS) for (psycho-)therapeutic settings.

Specific aspects of ALS for (psycho-)therapeutic settings	
Physical symptoms	– Muscle weakness – Dysarthria
Emotional symptoms/alterations	– Depression – Hopelessness – Feelings of helplessness – Pseudobulbar affect
Mental-cognitive challenges	– Confrontation with a fatal disease and own end of life – Adaptation to and acceptance of the new life situation – Computer-based means of communication – Decisions about permanent medical measures, including percutaneous endoscopic gastrostomy tube placement and assisted ventilation during disease progress

## Fundamentals of Psychological Interventions in Palliative Care

The high prevalence of mental burden like anxiety and depression, including hopelessness, despair, and demoralization in palliative patients demonstrate the need for effective psychological interventions integrated in palliative care concepts (30). Psychological interventions can address a wide spectrum of objectives in palliative care and together aim to reduce psychosocial distress and maintain quality of life in patients and their caregivers (31). These interventions intend to help the patient and family in coping with the fear of death and dying, managing anxiety, and reducing feelings of isolation, sadness, despair, and depression (32). Other psychological approaches address problems associated with changes of social roles and relationships, increasing dependence on others, the need to adjust to impaired functional status, and existential concerns, such as the search for meaning in life, hope, sense of dignity, grief, and spirituality (32). Clinical psychotherapeutic care for patients with progressing diseases comprises a variety of interventions and techniques, all of which have to be integrated into a multidisciplinary care plan. These include cognitive behavioral therapy, psychodynamic therapy, narrative interventions, relaxation and guided imagery, mindfulness-based interventions, meaning-focused interventions, art therapy, and dignity therapy (33).

### Psychotherapeutic Topics and Psychological Needs in Palliative Settings

The “psychotherapeutic work and goals in palliative care settings generally differ in several aspects from psychological interventions for patients with early or curative diseases or physically healthy individuals” (32).

In palliative settings, psychotherapeutic support starts with the diagnosis of the incurable disease. After communication of the diagnosis, patients often need time for reflection and room in which they can express their emotions. Because the delivery and communication of the diagnosis and its consequences is a crucial and emotionally relevant moment for the patient and his/her relatives, a psychologist in the medical team provides the patients with an additional opportunity to express their feelings and fears (34).

Furthermore, the time frame for psychotherapeutic interventions may be limited, especially in the case of ALS patients. Usually, “patients can be see” by the psychotherapist “only a few times, depending on their physical condition, the course of the disease,” and the setting (inpatient vs. outpatient). “The limited time has several implications for the development of a trustful and sustainable therapeutic relationship and psychotherapeutic treatment planning” (32). Treatment planning often depends on the stage and course of the disease and always has to be flexible enough to take into account spontaneous changes in the supportive care needs of patients or their caregivers. These and rapid changes in the course of the disease may place high demands on the clinical psychologist with regards to flexibility, empathy, and understanding of the patient’s situation (32).

“Treatment planning for patients with serious illnesses must also consider that communication with the patient and caregiver can be hampered by severe health conditions” (32). In ALS patients, poor articulation or even the inability to speak, and, in some cases, cognitive impairment and behavioral changes may significantly affect communication (35).

In addition, communication with the patient and caregiver can be compromised by unclear or divergent perceptions and prognostic awareness about the goals of treatment and the curability of the disease. Prognostic awareness contains multifactorial components, such as awareness of (i) the terminal nature of one’s illness, (ii) the purpose of treatment, or (iii) a shortened life expectancy (36). Palliative care patients show a wide range of prognostic awareness, reaching from 0 to 75% (37).

There are multiple reasons why patients report limited or inaccurate prognostic awareness including the lack of information given by physicians, such as incomplete understanding of the information, intentional or unintentional denial to accept the prognosis, and the phenomenon of “double awareness”: mixed states of awareness, hope for cure or hope for longer survival, despair or (partly) denial (36, 37).

Having this phenomenon of *double awareness* in mind, “the clinical psychologist is often faced with the difficult task of encouraging patients and caregivers to cope adaptively while promoting acceptance” (32). “Support for coping may focus on maintaining hope and quality of life, and reducing psychological stress. Acceptance may require that patients and caregivers face realistic treatment goals and treatment decisions, which themselves may negatively affect the psychosocial well-being of the patient and the family” caregivers. “The psychologist must be prepared to manage the emotional responses of the patient and the caregiver” and, finally, “clinical psychologists working in palliative care settings must be prepared to deal with” their own emotional reactions caused “by the closeness to death and dying,” helplessness, and existential or spiritual questions about the meaning of life and death (32).

## Introduction of the Psychotherapeutic Short-Term Intervention Based on Calm in ALS Patients

A psychotherapeutic short-term intervention has to face the above-mentioned characteristics in treatment of patients with advanced disease. Although psychological interventions are effective in reducing depression and anxiety and improving quality of life, the majority of randomized-controlled trials in physically ill patients are conducted in early stage cancer populations. Thus, data on psychological interventions in palliative care populations including life-threatening diseases other than cancer is scarce. Previous psychological studies in ALS patients have been mostly descriptive (38–40) and a cognitive behavioral therapy study failed because of slow recruitment and low demand for joint patient-caregiver therapy sessions (41).

“Managing Cancer and Living Meaningfully (CALM)” is a manualized, semi-structured, individual psycho-oncological short-term treatment to relieve distress and promote psychological well-being, which has been established by Rodin and colleagues (42, 43). It aims to reduce depression and fears about death and dying, to strengthen communication with the medical treatment team, and to improve the patients’ hope and meaning of life. It was developed based on empirical data, clinical observations, and leads back to different theoretical traditions, including relational theory (44), binding theory (45), and existential theory (46). Depending on the individual needs of the patient, CALM is built up by 3–8 sessions (duration about 45–60 min) over a period of 6 months. The sessions address four dimensions:

1. Symptom management and communication with healthcare providers
2. Changes in self and relations with each others
3. Spirituality, sense of meaning, and purpose
4. Thinking of the future, hope, and mortality

All dimensions are explored with every patient, but the order and extent of each dimension are adapted to the individual needs of the patient.

**Figure 1:**
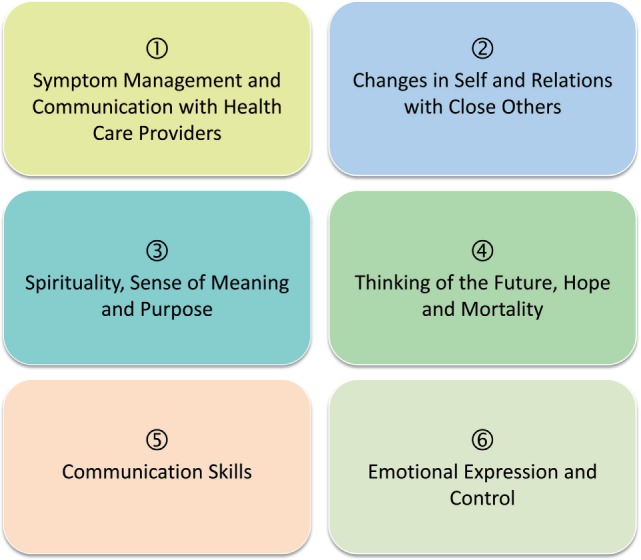
Dimensions of short-term psychotherapy ManagIng Burden in amyotrophic lateral sclerosis (ALS) and Living Meaningfully. Dimensions (1–4) of the short-term psychotherapy managing cancer and living meaningfully with the addition of the dimensions “Communication skills” (5) and “Emotional expression and control” (6) based on the specific symptoms and challenges of ALS patients.

For ALS patients, we consider it necessary to supplement the concept by two further dimensions based on the specific symptoms and challenges (Figure 1):

5. Communication skills
6. Emotional expression and control

Each participant’s primary caregiver (e.g., partner, adult son, or daughter) is offered the opportunity to participate in one or more of the therapy sessions, as deemed appropriate by the therapist and with the patient’s permission (43). During the course of treatment, different psychotherapeutic principles build the fundament of the therapy (47). One major aspect concerns the authenticity of the therapist and the development of a supportive relationship between therapist and patient. The capacity for mentalization/self reflection of the patient is supported by entertaining the possibility of multiple and complex psychological responses by the therapist to the expression of the patient. This strategy allows the patients to keep hope and accept the reality of their disease in parallel, so that they are able to plan future care and focus on new tasks. Crises due to disease progress and increased burden of symptoms may call for spontaneous changes of therapeutic aims. Therefore, content and timing of the psychotherapeutic sessions have to be adapted to the medical condition of the patient (48).

The therapist focuses on the emotional attachment of the patient. In detail, the changes in relationships to others as well as the resulting fears and sadness are explored. A dysbalance of relations is mostly seen in advanced stages of the disease, which results from high levels of dependence and the loss of autonomy (48).

During CALM therapy, the psychotherapist and the patient explore the meanings of the patient’s life history, including achievements and failures, as well as the disease itself. Thereby, the whole life trajectory of the patient, his/her aims, experience of suffering, and death/dying play important roles in the therapy. In the therapeutic contact, the therapist can explore how the patient makes sense of his or her situation and may help to facilitate meaning making as an adaptive way to cope with a situation beyond one’s control (48).

A pivotal element of the therapy is the willingness of the therapist to reflect his or her own philosophy and sense of meaning, and to face frightening topics such as mortality and suffering in order to encourage the patient to do the same. The therapeutic aim here is to allow the expression of sadness and fear regarding the progress of the disease and the confrontation with mortality, but to simultaneously support hope, courage, and engagement in the current moment (48). The therapist needs to be comfortable with the “non-expert” role and the “unsolvable” existential problems faced by patients with advanced disease.

For ALS patients, we identified the specific additional needs for training of communication skills, because many ALS patients develop a severe dysarthria and need to establish new forms of communication. We suggest a logopedic treatment included in the multimodal therapy of ALS patients to learn new communication strategies, e.g., with communication devices or eye-tracking systems.

In addition, we suggest a specific psychotherapeutic focus on emotional expression and control because of a significant number of ALS patients suffer from depression and/or pseudobulbar affect.

Feasibility and effectiveness of CALM to reduce emotional distress and promote psychological well-being and growth have been tested in qualitative studies and in a Phase 2 trial in patients with advanced cancer (42, 43, 48). In the qualitative studies, no patient-reported risks or concerns have been found (42). Rather, participants described five main benefits of the intervention, which are (i) “a safe place to process the experience of advanced cancer, (ii) permission to talk about death and dying, (iii) assistance in managing the illness and navigating the healthcare system, (iv) resolution of relational strain and (v) an opportunity” to be seen as a whole person “within the healthcare system” (42).

The qualitative results were also supported by quantitative findings: results of the phase 2 trial showed that depressive symptoms and death anxiety decreased significantly under CALM treatment and that spiritual well-being increased in the 3- and 6-month follow-up assessments (43).

Given empirical data on the effectiveness of CALM among cancer patients, this concept might also be useful in patients with other advanced diseases. This might be especially true for patients with ALS, who are, similar to advanced cancer patients, confronted with a mostly fast progressing disease, mortality, and finiteness of life.

Based on the above considerations, we posit that CALM might be an effective psychotherapeutic treatment in ALS patients with the addition of the dimensions “Communication skills” and “Emotional expression and control”. To prove applicability and efficacy of this therapeutic concept “mi-BALM” (ManagIng Burden in ALS and Living Meaningfully) for ALS patients, we propose a trial on this semi-structured, individual psychotherapeutic short-term intervention in ALS patients. Demonstration of efficacy of mi-BALM in ALS would fulfill a strong need for improving the physical and psychological quality of life in this patient group and may be beneficially implemented in the interdisciplinary therapy of ALS patients. Having established this therapy, web- or telephone-based forms of this treatment could be developed in order to ensure dissemination of this therapy in patients with advanced stages of diseases or patients from rural areas.

## Author Contributions

MO, AM, and JC participated in the design of the perspective concept. MO, PE, JC, and AM participated in writing process. MO, PE, and AM participated in the creation of figures and tables. All authors have reviewed the manuscript.

## Conflict of Interest Statement

The authors declare that the research was conducted in the absence of any commercial or financial relationships that could be construed as a potential conflict of interest.
